# Advancing Nutritional Science: Contemporary Perspectives on Diet’s Role in Metabolic Health and Disease Prevention

**DOI:** 10.3390/nu17091414

**Published:** 2025-04-23

**Authors:** Iñaki Elío

**Affiliations:** 1 Research Group on Foods, Nutritional Biochemistry and Health, Universidad Europea del Atlántico, 39011 Santander, Spain; inaki.elio@uneatlantico.es; 2 Faculty of Health Sciences, Universidad Internacional Iberoamericana, Arecibo, PR 00613, USA; 3 Department of Health, Nutrition and Sport, Universidad Internacional Iberoamericana, Campeche 24560, Mexico

## 1. Introduction

This Special Issue of Diet and Nutrition: Metabolic Diseases showcases cutting-edge research exploring the intersection between nutrition, dietary patterns, and public health. The contributions in this collection involve both fundamental and applied research, offering new insights into how nutrition can combat the growing global burden of non-communicable diseases [1]. The studies in this issue emphasize the critical role that diet plays in promoting metabolic health, preventing chronic diseases, and improving overall quality of life.

In recent years, nutrition has become a central focus in global health efforts, with a growing body of evidence demonstrating its impact on both individual and population-level outcomes [2,3]. This Special Issue encompasses several key themes, including the role of dietary interventions in managing metabolic disorders, the importance of nutrient timing and quality, and the broader implications of sustainable dietary practices.

## 2. An Overview of Published Articles

The study by Cubas-Basterrechea et al. [4] examined the association between vegetable intake and metabolic syndrome (MetS) in 264 older adults aged 65–79 years. Only 17% met the recommended daily intake of ≥2 servings (400 g) of vegetables. Adequate intake was linked to a 19% lower MetS prevalence (24.4% vs. 43.4%, *p* < 0.05), while inadequate consumption significantly increased MetS risk (OR: 2.21; *p* = 0.035). The authors emphasized the need to encourage vegetable consumption in older populations to reduce MetS prevalence.

Gwizdak et al. [5] investigated the dietary habits and nutritional knowledge of 297 women aged 18–45 with thyroid disorders, focusing on the impact of education level. Hypothyroidism and Hashimoto’s disease were most common in younger women (18–25 years). Higher education correlated with better awareness of protein, carbohydrate roles, and nutrient–drug interactions (*p* < 0.01), as well as healthier cooking practices. Low water intake was linked to comorbidities like insulin resistance and cardiovascular disease (*p* < 0.01). The authors highlighted significant gaps in dietary knowledge and emphasized the need for tailored educational interventions to improve thyroid disorder management.

An study with mice Huang et al. [6] explored short-term zinc supplementation (30–90 ppm zinc sulfate in drinking water for one week) in C57/BL6J male mice. Despite no changes in body weight or food intake, zinc reduced visceral fat deposition and adipocyte size by **enhancing lipolysis** (↑ *Atgl*, *Hsl*, p-HSL, and PPARγ) and **suppressing lipogenesis** (↓ FASN protein). However, zinc increased serum insulin levels (*hyperinsulinemia*) and adipose inflammation markers (↑ *F4/80*, *Tnfa*). The authors propose short-term zinc supplementation as a potential strategy for fat reduction but caution its inflammatory side effects, suggesting future studies on intermittent dosing protocols.

The study by Sprenghini et al. [7] retrospectively examined adrenoleukodystrophy (X-ALD), a metabolic disorder caused by the dysfunctional peroxisomal beta-oxidation of very-long-chain fatty acids (VLCFAs) and the consumption of a Mediterranean diet low in VLCFAs in 36 patients and carriers at baseline and one year following the introduction of dietary restrictions The authors concluded that a diet low in VLCFAs is highly recommended as a supplement to treatment because it effectively lowers plasma VLCFA levels.

Another noteworthy contribution is the study by Liu et al. [8], which investigated the impact of dietary patterns on metabolic syndrome in young adults and how physical activity modulates this effect. A cross-sectional study was conducted at a health management center in Tianjin, China, from September 2022 to March 2023. Participants aged 18–35 years were recruited using convenience sampling. No significant associations were found for the Sugar–Processed and Alcohol–Meat patterns. Subgroup analysis revealed that the Legume–Nut pattern increased the risk of metabolic syndrome among those with irregular physical activity, whereas the Egg–Vegetable pattern decreased the risk. These findings highlight the significant influence of dietary patterns on the risk of metabolic syndrome in young adults and the modifying effect of regular physical activity, underscoring the need for targeted dietary and lifestyle interventions to prevent metabolic syndrome in this population.

Khoshkerdar et al. [9] examined how pregnancy causes significant changes in the mother’s physiology and homeostatic regulation in rats. Pregnancy-related disruptions in maternal adaptations are linked to poor maternal diet; however, the impact of paternal diet on maternal health is less well established. This illustrates how patterns of maternal metabolism and gestation-related modifications to the mother’s physiology can be impacted by the paternal diet at the time of mating.

The study by Rauch et al. [10] examined how administering a combination of ketone diester (KE) and medium-chain triglyceride (MCT) oil, known as KEMCT, to WAG/Rij rats eliminated the rise in blood glucose levels brought on by isoflurane anaesthesia and extended the recovery period. These findings imply that EKSs may be useful in reducing the adverse metabolic effects of isoflurane, including hyperglycaemia, in both sexes when administered as adjuvant therapy.

A laboratory study by Torrens et al. [11] showed that consuming carbohydrates prior to exercise significantly reduces the symptoms of McArdle disease (IWMD) and increases exercise tolerance in the early stages of exercise. Information regarding the type and quantity of carbohydrates consumed before exercise was gathered using an online survey method. Merely 17.5% of participants said that eating carbohydrates prior to exercise reduced or alleviated their symptoms of MD.

In the sixth article, Xu et al. [12] investigated how to improve the postprandial glycaemic response (PPGR) and postprandial insulin response (PPIR) after consuming biscuits made with 30% wheat flour and autoclaved BSG (ABSG) or fermented BSG (FBSG) in people with metabolic syndrome (MetS). The impact on the subjective appetite response, breath hydrogen (H2) and methane (CH4) concentrations, and postprandial lipid panel was also investigated. In comparison to the control group, the insulin level decreased 180 min after consuming FBSG (*p* = 0.051) and ABSG (*p* = 0.010). However, subjective appetite response, breath H2 and CH4 content, and postprandial lipid panel did not differ. In summary, eating biscuits with BSG can reduce the PPGR, and adding fermented BSG provides an additional benefit in controlling PPGR.

The study by Bugi et al. [13] used the “Food Neophobia Scale” (FNS) to measure neophobia in 34 individuals with phenylketonuria, who had previously received a diagnosis, and a control group that ranged in age from 7 months to 40 years. The statistics showed that 70.57% of the control group and 61.76% of PKU patients were neophobic. The current age of PKU patients, the time between birth and PKU diagnosis, and the educational attainment of their parents were all linked to food neophobia.

The study by Li et al. [14] investigated various stages of metabolic dysfunction-associated steatosic liver disease (MASLD) by prolonging the incubation period of human precision-cut liver slices (PCLSs). To develop MASLD, healthy human PCLSs were cultivated for up to 96 h in a medium supplemented with high levels of sugar, insulin, and fatty acids. Hepatic steatosis, which is defined by the accumulation of intracellular fat, was seen in PCLSs. A time-dependent effect on lipid metabolism seemed to be involved in the development of hepatic steatosis, with fatty acid absorption and storage initially increasing and lipid oxidation and secretion later decreasing. The authors concluded that a strong ex vivo model for MASLD can be created by incubating human PCLSs for an extended period, making it easier to find and assess possible treatment options.

In the ninth article, Alonso-Allende et al. [15] reviewed the main effects of inulin on human metabolic health, with a particular focus on the mechanisms of action of this prebiotic. Inulin supplementation contributes to body weight and BMI control, reduces blood glucose levels, improves insulin sensitivity, and reduces inflammation markers, mainly through the selective favouring of short-chain fatty acid (SCFA)-producing species from the genera Bifidobacterium and Anaerostipes. This review indicated that consuming inulin improves the gut microbiota and creates compounds through fermentation, which have beneficial metabolic effects.

The potential of royal jelly (RJ), a naturally occurring bee product that is high in bioactive components, was examined in the article by El Seedi et al. [16] as an alternate approach to treating metabolic illnesses. RJ demonstrates antimicrobial, estrogen-like, anti-inflammatory, hypotensive, anticancer, and antioxidant properties, among others. RJ’s function in regulating immunological responses, boosting anti-inflammatory cytokines, and inhibiting important inflammatory mediators has been highlighted in recent studies. Despite these encouraging results, more research is required to fully comprehend the processes underlying RJ’s therapeutic effects.

In the last article, Gao et al. [17] provided a comprehensive review of the application and mechanisms of probiotic-mediated gut microbiota homeostasis in skin care and offered a theoretical basis for the application of probiotics in skin care. Although the preventive properties and activities of topical probiotics helped to preserve skin homeostasis, their drawbacks and restrictions resulted in inflammatory skin disorders that are challenging to fully treat with topical probiotics. The effectiveness and side effects of internal probiotic formulations for the treatment of wounds, psoriasis, acne, atopic dermatitis, and numerous other skin issues are being investigated in several clinical trials.

## 3. Several Key Themes Emerge from the Research Presented

**1. Dietary Patterns and Metabolic Health**: In this issue, a cross-sectional study examines how food patterns affect metabolic syndrome in young individuals while simultaneously examining how physical activity can modulate this condition [8]. The intake of ≥2 servings (400 g) of vegetables lower the risk of MetS in a 19% in older adults aged 65–79 years [4]. A cross-sectional study stablished that Hashimoto’s disease were most common in younger women (18–25 years), higher education correlated with better awareness of protein, carbohydrate roles, and nutrient–drug interactions [5]. These studies highlight the importance of tailored dietary strategies to improve metabolic and thyroid health across age groups. For young adults, future research should focus on how physical activity can optimize the benefits of healthy dietary patterns, such as the Egg-Vegetable diet, in reducing metabolic syndrome risk. Additionally, efforts to enhance vegetable intake in older adults and nutritional education in younger women with thyroid disorders are crucial for improving overall health outcomes.

**2. Innovative Dietary Approaches**: The studies in this issue explore novel nutritional interventions, such as the use of brewers’ spent grain in biscuits to improve glycaemic response in metabolic syndrome patients [12]. These findings highlight the potential of repurposing food industry by-products to yield health benefits, which is a growing trend in sustainable nutrition.

**3. Personalized Nutrition**: The research on carbohydrate ingestion for individuals with McArdle disease underscores the importance of tailored nutritional strategies for rare metabolic disorders [11]. This research emphasizes the need for condition-specific dietary recommendations, aligning with broader trends in personalized nutrition.

**4. Gut–Metabolic Health Connection**: Multiple studies explore the intricate relationship between gut health and metabolic function. The review on probiotics and skin health via the gut–skin axis demonstrates the significant impacts of gut microbiota on overall health [17], supporting growing evidence of the gut microbiome’s role in metabolic health.

**5. Nutritional Management of Rare Disorders**: Articles addressing conditions such as phenylketonuria [13] and adrenoleukodystrophy [7] provide valuable insights into the dietary management of rare metabolic diseases, highlighting the crucial role of nutrition in these complex conditions.

**6. Metabolic Adaptations and Diet**: Research on maternal adaptations to pregnancy and the impact of paternal diet offers intriguing perspectives on how nutrition influences metabolic health across generations [9].

**7. Novel Research Models**: The development of human precision-cut liver slices as a model for metabolic dysfunction-associated steatotic liver disease showcases an innovative approach to studying metabolic conditions and screening potential interventions [14].

**8. Nutraceutical Potential**: Reviews on compounds like inulin [15] and royal jelly [16] explore the therapeutic potential of natural products in addressing metabolic disorders, opening avenues for future research and potential interventions. The short-term zinc supplementation might reduce fat deposition in mice [6].

**9. Metabolic Effects of Anesthesia and Ketone Supplements**: This research highlights the potential of ketone supplements in mitigating the metabolic side effects of anesthesia, contributing to our understanding of metabolic adaptations in medical interventions [10].

## 4. Conclusions

This Special Issue not only enhances our understanding of the complex connections between nutrition, food, and metabolic disorders but also explores areas for future study and therapeutic application. The diverse topics covered in this issue illustrate the complexity of metabolic health and the importance of nutrition in preserving and restoring it.

We extend our sincere gratitude to the editorial team for their dedicated efforts in realizing this Special Issue, the peer reviewers for their insightful feedback, and all the authors for their invaluable research contributions.
